# Fuzheng Huayu tablets for treating pulmonary fibrosis in post-COVID-19 patients: a multicenter, randomized, double-blind, placebo-controlled trial

**DOI:** 10.3389/fphar.2025.1508276

**Published:** 2025-03-11

**Authors:** Fei Jing, Wei Wang, Jia Ke, Tingrong Huang, Bo Jiang, Qiwu Qiu, Jihan Huang, Songhua Zhan, Wei Zhang, Hui Wu, Wen Su, Jiawen Feng, Yuan Peng, Zhimin Zhao, Feng Xing, Chenghai Liu

**Affiliations:** ^1^ Institute of Liver Diseases, Shuguang Hospital affiliated to Shanghai University of Traditional Chinese Medicine, Shanghai, China; ^2^ Shandong University of Traditional Chinese Medicine Affiliated Hospital, Jinan, China; ^3^ Hubei University of Chinese Medicine, Wuhan, China; ^4^ Department of Respiratory, Hubei Provincial Hospital of Traditional Chinese Medicine, Wuhan, China; ^5^ Huangshi Hospital of Traditional Chinese Medicine, Huangshi, China; ^6^ Department of Traditional Chinese Medicine, Wuhan Third Hospital, Wuhan, China; ^7^ Department of Infectious Diseases, Jingmen First People’s Hospital, Jingmen, China; ^8^ Center for Drug Clinical Research, Institute of Interdisciplinary Integrative Medicine Research, Shanghai University of Traditional Chinese Medicine, Shanghai, China; ^9^ Department of Radiology, Shuguang Hospital affiliated to Shanghai University of Traditional Chinese Medicine, Shanghai, China; ^10^ Department of Respiratory, Shuguang Hospital affiliated to Shanghai University of Traditional Chinese Medicine, Shanghai, China; ^11^ Department of Traditional Chinese Medicine, Wuhan Integrated TCM and Western Medicine Hospital, Wuhan, China; ^12^ Office of Academic Research, Wuhan Integrated TCM and Western Medicine Hospital, Wuhan, China; ^13^ Shanghai Key Laboratory of Traditional Chinese Clinical Medicine, Shanghai, China; ^14^ Key Laboratory of Liver and Kidney Diseases, Ministry of Education, Shanghai, China

**Keywords:** Fuzheng Huayu tablet, pulmonary fibrosis, pulmonary function, COVID-19, randomized controlled trial

## Abstract

**Background:**

Effective therapies for pulmonary fibrosis caused by coronavirus disease (COVID-19) and other etiologies are lacking. Our previous studies demonstrated that Fuzheng Huayu tablet (FZHY), a traditional Chinese medicine known for its anti-liver fibrotic properties, can improve lung function in patients with chronic obstructive pulmonary disease and attenuate bleomycin-induced pulmonary fibrosis in rats.

**Purpose:**

This study aimed to evaluate the efficacy and safety of FZHY in post-COVID-19 pulmonary fibrosis.

**Methods:**

A multi-center, randomized, double-blind, placebo-controlled clinical trial was conducted to evaluate the efficacy of a 24-week treatment with FZHY, combined with vitamin C and respiratory function rehabilitation, for treating pulmonary fibrosis in discharged convalescent COVID-19 patients. The primary outcome was the regression rate of pulmonary fibrosis assessed by the high-resolution computed tomography scores and lung function improvement (forced vital capacity [FVC], forced expiratory volume in one second [FEV1], and FEV1/FVC) after 24 weeks. Secondary outcomes included the 6-min walk distance, improvement in pulmonary inflammation, clinical symptoms, and quality of life.

**Results:**

This study included 142 patients, who were randomized to the FZHY (n = 72) and placebo groups (n = 70). By week 24, the regression rates of pulmonary fibrosis in the FZHY and placebo groups were 71.2% and 49.2%, respectively (p = 0.01). Limited spirometry data revealed higher FEV1/FVC in the FZHY group than in the placebo group at week 8 ([87.7 ± 7.2] % vs. [82.7 ± 6.9] %; p = 0.018). The regression rates in pulmonary inflammation in the FZHY and placebo groups were 83.8% and 68.8%, respectively (p = 0.04). At week 4, the increase in 6-min walking distance was greater in the FZHY group than in the placebo group ([41.4 ± 64.1] m vs. [21.8 ± 50.3] m; p = 0.05). However, no significant differences were observed between the groups in the improvement rate of clinical symptoms, quality of life-BREF, patient health questionnaire-9, or generalized anxiety disorder-7 scores (p > 0.05). No drug-related adverse events were reported in the FZHY group.

**Conclusion:**

FZHY attenuates post-COVID-19 pulmonary fibrosis, with good safety profiles.

**Clinical Trial Registration:**

https://clinicaltrials.gov/study/NCT04279197, identifier NCT04279197.

## Introduction

Pulmonary fibrosis, a progressive interstitial lung disorder characterized by the proliferation of lung fibroblasts, extracellular matrix deposition, and infiltration of airway inflammatory cells, is a common complication of numerous lung diseases that can induce restrictive ventilatory dysfunction and impaired diffusion function (Wuyts et al., 2013). Thoracic imaging findings reveal various degrees of cord- or grid-like shadowing.

Viral infections, such as severe acute respiratory syndrome (SARS) and cytomegalovirus infections, contribute to pulmonary fibrosis (Venkataraman et al., 2017). The coronavirus disease-19 (COVID-19) pandemic caused by the severe acute respiratory syndrome coronavirus 2 (SARS-CoV-2) outbreak in late 2019 has affected over 760 million people worldwide. Some COVID-19 patients experience varying degrees of sequelae during recovery (Zhao et al., 2020). Wang evaluated the pathological findings in the lungs of 12 deceased COVID-19 patients, which exhibited diffuse alveolar damage, interstitial fibrosis, and exudative inflammation (Liu et al., 2020). Pulmonary dysfunction and abnormal imaging findings, such as ground-glass opacities and bands of parenchymal fibrosis, were observed in convalescent COVID-19 patients (Shaw et al., 2021). Additionally, pulmonary fibrosis was reported in 4.9% of 287 convalescent COVID-19 patients (Kamal et al., 2021).

Therefore, further investigation on post-COVID-19 pulmonary fibrosis is warranted. Currently, only pirfenidone and nintedanib have been approved for the treatment of idiopathic pulmonary fibrosis (Martinez et al., 2017). Pirfenidone and nintedanib delay the progression of pulmonary fibrosis but do not significantly improve the overall survival and prognosis. Moreover, both drugs are expensive and associated with substantial side effects such as gastrointestinal reactions and liver dysfunction. (Cottin and Maher, 2015; King et al., 2014; Flaherty et al., 2018). The efficacy of pirfenidone and nintedanib for treating pulmonary fibrosis in patients with COVID-19 is still under investigation.

Pulmonary fibrosis is a complex condition involving multiple targets that is difficult to treat with a single agent. Traditional Chinese Medicine (TCM), with its multi-component, multi-targeted approach, and extensive experience in treating chronic lung diseases, offers a practical approach for developing anti-pulmonary fibrosis drugs to meet the emerging clinical needs. Fuzheng Huayu tablet (FZHY), a plant-based formulation composed of six herbs, has been approved by China’s National Medical Products Administration in 2003 for the treatment of liver fibrosis. FZHY has good quality control with few adverse reactions. FZHY can reverse hepatitis B-induced liver fibrosis by attenuating hepatocyte inflammation, inhibiting hepatic stellate cell activation, and regulating hepatic matrix metabolism (Wang et al., 2018; Liu et al., 2009). A clinical trial showed that FZHY improved pulmonary function in Chinese patients with chronic obstructive pulmonary disease (COPD) patients. Moreover, FZHY attenuates pulmonary inflammation and regresses fibrosis in bleomycin (BLM)-induced pulmonary fibrosis in rats (Tan et al., 2007). Given the potential similarities in the pathological mechanisms between pulmonary fibrosis in patients with COVID-19 and fibrotic disorders in other organs, we conducted this clinical trial in which patients with post-inflammatory pulmonary fibrosis due to COVID-19 in the recovery period were randomized to receive either FZHY or a placebo. Given that COVID-19 is a novel disease and the lack of specific treatments for post-inflammatory pulmonary fibrosis, a placebo parallel-controlled study was performed. This study aimed to evaluate the efficacy and safety of FZHY in treating pulmonary fibrosis in convalescent patients with COVID-19.

## Methods

### Trial design

This multi-center, double-blind, randomized, placebo-controlled clinical trial was conducted at five centers in China, including Hubei Provincial Hospital of Traditional Chinese Medicine, Huangshi Hospital of Traditional Chinese Medicine, Wuhan Third Hospital, Jingmen First People’s Hospital, Wuhan Integrated TCM & Western Medicine Hospital. Eligible patients were recruited and randomly assigned to the FZHY group and the placebo group in a ratio of 1:1 using the interactive web response system (IWRS). This study was approved by the ethics committee of Shuguang Hospital Affiliated to Shanghai University of Traditional Chinese Medicine (2020-798-05-01) and the ethics committee of each subcenter. The protocol (Jing et al., 2021) was designed in accordance with the guidelines outlined in Good Clinical Practice and the Declaration of Helsinki. All patients have signed informed consent forms. This study adopts the method of competitive enrollment.

### Participants

The diagnostic criteria for suspected and diagnosed COVID-19 cases were based on the *Diagnosis and Treatment Guideline for COVID-19 (Trial seventh Edition)* released by the National Health Commission of the People^’^s Republic of China (Diagnosis and Treatment Protocol for COVID-19, 2020). The inclusion criteria were as follows: 1) discharged patients with a confirmed diagnosis of COVID-19; 2) two consecutive negative SARS-CoV-2 RNA tests in respiratory or blood samples by real-time fluorescent polymerase chain reaction; 3) evidence of pulmonary fibrosis on computed tomography (CT) performed within 7 days; 4) age 18–70 years. The exclusion criteria were as follows: 1) patients who had undergone lung surgery, such as lung transplantation and lung resection, which affects lung function; 2) dependence on mechanical ventilation; 3) presence of chronic lung diseases that impair lung function, such as COPD; 4) conditions affecting cardiac function, such as pulmonary hypertension, heart failure, and pacemaker installation; 5) diseases affecting survival; 6) resting heart rate >120 beats/min; 7) systolic blood pressure >180 mmHg and diastolic blood pressure >100 mmHg; 8) history of myocardial infarction or unstable angina within the previous month; 9) severe obesity (body mass index [BMI] > 30 kg/m^2^); 10) allergy to pharmaceutical ingredients involved in the treatment regimen; 11) pregnant or breastfeeding women; 12) inability to complete the efficacy evaluation questionnaires; 13) patients enrolled in other clinical trials within 1 month prior to enrolment in this trial or simultaneous participation in other clinical trials; 14) poor compliance. Informed consent was obtained from all patients.

### Interventions

The FZHY group received FZHY tablets (0.4 g/tablet, Shanghai Huanghai Pharmaceutical Co., Ltd. Lot number S200301, expiry date 2023/02) during the treatment period, and 1.6 g (4 tablets) was administered orally approximately 30 min after meals three times/day for 24 consecutive weeks.

FZHY tablet refers to the herbal extraction, which has been licensed (Identifier No. Z20050546) by Chinese National Medical Products Administration and comprises Danshen (*Salvia miltorrhiza* Bunge [Lamiaceae; radix]), Chongcao (artificialis fermentation cordyceps; mycelia), Taoren (*Prunus persica* (L.) Batsch [Rosaceae; fruit]), Jiaogulan (*Gynostemma pentaphyllum* (Thunb.) Makino [Cucurbitaceae; whole herb]), Songhuafen (*Pinus massoniana* Lamb. [Pinaceae; pollen]), and Wuweizi (*Schisandra chinensis* (Turcz.) Baill. [Schisandraceae; fruit]). The species of ingredients are covered in Chinese pharmacopoeia. All the raw materials for FZHY come from the medicinal material planting and harvesting bases according to Good Agricultural and Collection Practices requirement in definite places, and identified for their origin, morphological, microscopic, physical, chemical characteristics, as well as the DNA barcode identification in order to guarantee the authenticity and quality of medicinal materials.

The manufacturing process is conducted as follows: For the preparation of FZHY extraction, 666g of Danshen, 500g of Jiaogulan, 334g of Chongcao, 166g of Taoren, 166g of Songhuafen and 166g of Wuweizi are weighed up. Danshen, Taoren and Jiaogulan are first mixed with appropriate amount of water to decoct twice, 2 h for the first time and 1.5 h for the second time. The decoctions are then combined and left to stand for 24h to allow the supernatant to concentrate until a relative density of about 1.20 (50°C–55°C). This is then cooled, and alcohol (95%) is added while slowly shaking up until the alcohol content reaches 70%. It is then cooled again, filtrated and concentrated until the relative density reaches 1.3–1.4 at 50°C–55°C. Drying takes place by decompression to obtain the dry extract. Then, Chongcao and Wuweizi are weighed up, reflux extraction with alcohol twice, 2 h for the first time and 1.5 h at the second time. The extract solution is combined, filtrated and concentrated until the relative density reaches 1.3–1.4 at 50°C–55°C. The alcohol is recovered and dried by decompression to get the dry extract. Similarly, Songhuafen is soaked in 50% alcohol at 40°C twice, 4 h for the first time and 2 h for the second time. The extract solution is combined, filtrated and concentrated until the relative density reaches 1.3–1.4 at 50°C–55°C The alcohol is recovered, dried and decompressed to obtain the dry extract. Those three dry extracts are mixed and dried as the FZHY extract powder to reserve. Then pharmaceutical excipients are added to the FZHY extract powder and the tablets are prepared directly by compression. It can be compressed to 1000 tablets (0.4g each).

To control the quality of the FZHY extracts, the fingerprint spectrum was established using high performance liquid chromatography (HPLC) method. Assay validation was performed according to the United States Food and Drug Administration bio-analytical method validation guideline. The contents of adenosine and danshensu were 2.5 mg/g and 8.04 mg/g in the extracts respectively, according to quality inspection report from the Shanghai Sundise Chinese Medicine Technology Development Co., Ltd (Shanghai, China). The formulation, standard manufacturing process, chemical component content, and multicomponent assay (fingerprinting) of the FZHY tablets are available in the Supplementary Material (Supplementary Table S1; Supplementary Figure S1; Supplementary Table S2; Supplementary Figure S2).

The placebo group received four placebo tablets 3 times a day orally for 24 weeks. The placebo, which was also manufactured by Shanghai Huanghai Pharmaceutical Co., Ltd. (Lot number S200202, expiry date 2023/01), consisted of a mixture of sucrose, starch, and sodium carboxymethyl starch to mimic the tablet shape of FZHY. Additionally, this mixture contained 1% FZHY to impart odor similar to that of the FZHY tablet.

All patients received basic treatment of respiratory function rehabilitation training according to the “COVID-19 patient rehabilitation program” (COVID-19, 2020) issued by the National Health Commission of the People’s Republic of China along with vitamin C tablets (0.2 g/dose, three times/day, orally, Shanghai Sine Tianping Pharmaceutical Co., Ltd.).

### Outcomes

The primary outcomes of the trial were the regression rate of pulmonary fibrosis after 24 weeks of treatment, as assessed by HRCT scores, and the improvement in pulmonary function after 24 weeks, as measured by spirometry (FVC, FEV1, and FEV1/FVC). The HRCT scores were independently assessed by two radiologists specializing in chest imaging and one respiratory physician. Disagreements were resolved through discussion.

Chest CT scans were performed using a spiral CT scanner with 64 rows or more. Scan parameters: tube voltage, 120 kV; smart tube current. Layer thickness: 1 mm. Reconstruction interlayer spacing: 1 mm. Matrix: 512 × 512. Method: Patients were positioned supine with their arms raised, instructed to hold their breath, and the scans were acquired at the end of deep inspiration. An inorganic shelf tilt was used, and the scan orientation was recommended to be 1 cm below the diaphragm, extending to the lung apex. No more than one tomogram was allowed to be missing between layers, and a series allowed up to three missing tomograms. Storage format: nondestructive compression, standard DICOM. For evaluation, the bilateral lobes were divided into six zones: the posterior and anterior segments of the upper lobe tip of the left lung were combined into one zone (referred to as the “upper left” zone), and the lingual segment of the upper lobe of the left lung was combined into one zone (the “middle left” zone). The lower lobe of the left lung and the upper, middle, and lower lobes of the right lung were designated as individual zones the “lower left,” “upper right,” “middle right,” and “lower right” zones. Each lung region was scored semi-quantitatively for inflammation (mainly lesions characterized by ground-glass opacities, solid lung changes, or a mixture of both) and fibrosis (grid-like or cord-like changes). Scores were assigned to the nearest 10% or within 10%, based on the extent of lesion involvement as follows: no inflammatory or fibrotic lesions within the lung region were scored as 0% and involvement of all lung regions were scored as 100%. The mean values for the percentages of lung inflammatory and fibrotic involvement were calculated, with higher mean scores indicating more severe lesions (Xaubet et al., 1998). A decrease in these scores was considered an improvement.

Secondary outcomes included the 6-min walk distance and improvements in pulmonary inflammation, clinical symptoms, and quality of life after 24 weeks of treatment. The 6-min walk distance was conducted and recorded by trained investigators. The regression rate of pulmonary inflammation was determined by the HRCT scores, which were assessed by the same radiologists and respiratory physician. Clinical symptoms, such as shortness of breath, cough, fatigue, insomnia, sweating, poor appetite, and diarrhea, were reported subjectively by the patients. Quality of life evaluations were obtained using questionnaire forms, including the quality of life-BREF (QOL-BREF) (WHO Quality of Life-BREF, 2020), patient health questionnaire-9 (PHQ-9) (Kroenke et al., 2001), and generalized anxiety disorder-7 (GAD-7) scores (Spitzer et al., 2006). During the treatment period, six follow-up visits were scheduled, once every 4 weeks.

The trial implemented appropriate measures to monitor adverse events (AEs), including the observation of vital signs, laboratory tests, and recording of concomitant medications. All AEs, regardless of their severity, were documented to assess the safety of FZHY.

### Sample size

Because COVID-19 is an emergent disease, the available references were limited at the time of protocol design; therefore, we used a placebo-controlled parallel trial design with a planned sample size of 160 patients (80 in each group).

### Randomization and blinding

A dynamic random approach using minimization was used in this study. After obtaining informed consent and completion of screening assessments to confirm the eligibility criteria, random numbers and medications were assigned in a 1:1 ratio using the IWRS. The randomization table generated by the IWRS was sealed in opaque envelopes.

All the investigators, patients, data managers, and data supervisors were blinded to the study protocols. The patients were automatically assigned unique random numbers through the IWRS, which remained consistent until the end of the trial. During the trial, researchers used these randomized numbers to distribute the corresponding drugs to the patients. Data managers, data supervisors, and researchers recorded patient data solely based on the random numbers.

### Statistical methods

Measurement data that followed a normal distribution were presented as the mean and standard deviation (SD), and non-normally distributed data were described by median and interquartile range (IQR). Comparisons were performed using either t-tests or rank sum tests, depending on the normality of the distribution. Count data were expressed as the number (percentage) of cases, and the chi-square test or Fisher’s exact test was used for comparison. All statistical analyses were performed using the SAS software version 9.4 (SAS Institute, Cary. NC, USA). Two-sided tests were used for all statistical tests, and a *P*-value of ≤0.05 was considered statistically significant.

The datasets included the full analysis set (FAS), per-protocol set (PPS), and safety analysis set (SS). For the primary efficacy measure, if data were missing, the previous outcome carried forward method was used, according to the intention-to-treat analysis (ITT analysis), with no carry forward for the secondary efficacy measures. The FAS was used as the primary dataset. Patients lost to follow-up were classified as ineffective.

## Results

**FIGURE 1:**
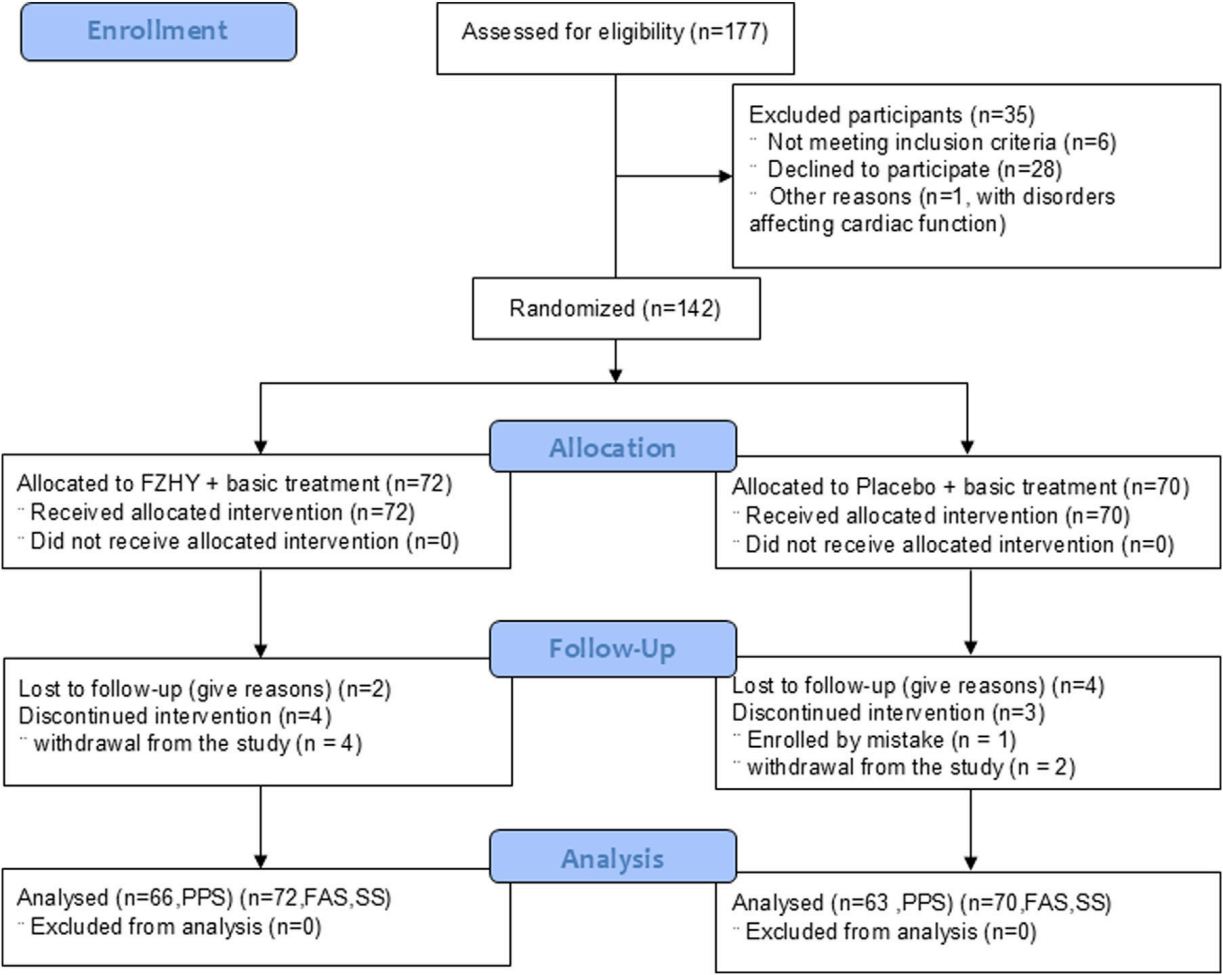
Flowchart FZHY: Fuzheng Huayu; PPS: per protocol set; FAS: full analysis set; SS, safety analysis set.

This study initially enrolled 177 eligible patients between 18 March 2020 and 17 March 2021. After excluding 35 patients (patient withdrawal, radiologic normality on chest HRCT, or disorders affecting cardiac function), 142 were included in the FAS (72 in the FZHY group and 70 in the placebo group). Additionally, six patients in the FZHY group and seven patients in the placebo group dropped out and were excluded from the PPS (Figure 1). The distribution of participants in each center is listed in Supplementary Table S3.

Clinical characteristics and baseline data showed that most patients were aged ˃ 45 years, with ˂ 50% of men in the total sample. Of the 142 patients with pulmonary fibrosis, 132 showed unabsorbed pulmonary inflammation. No significant differences in age, sex, disease duration (days from symptom onset to enrolment), clinical classification, comorbidities, 6-min walk distance, quality-of-life evaluations, and clinical symptoms were observed between the FZHY and placebo groups (p > 0.05, Table 1). Additionally, concomitant medications did not differ significantly between the two groups (Supplementary Table S4).

**TABLE 1:** Baseline Characteristics (Full analysis set).

	FZHY group (n = 72)	Placebo group (n = 70)	*p-*value
Age (years, x ± s)	57.6 ± 8.69	58.2 ± 11.54	0.714
Males (n, %)	27 (37.5%)	32 (45.7%)	0.321
BMI (kg/m^2^, x ± s)	24.39 ± 3.128	24.33 ± 2.897	0.903
Smoking history (n, %)	5 (6.9%)	8 (11.4%)	0.354
Alcohol consumption (n, %)	8 (11·1%)	11 (15·7%)	0.421
Temperature (°C, x ± s)	36.46 ± 0·234	36.48 ± 0.250	0.692
Respiratory rate (bpm, x ± s)	20.1 ± 2.56	20.5 ± 2.41	0.288
Heart rate (bpm, x ± s)	78.8 ± 10.32	80.6 ± 10.63	0.321
Systolic blood pressure (mmHg, x ± s)	126.3 ± 12.95	130·9 ± 16.14	0.062
Diastolic blood pressure (mmHg, x ± s)	79.4 ± 8.63	80.4 ± 10.60	0.562
Clinical classification (n, %)			
Mild	5 (7.0%)	6 (8.6%)	0.136
Common	39 (54.9%)	47 (67.1%)	
Severe	21 (29.6%)	13 (18.6%)	
Critical	6 (8.5%)	4 (5.7%)	
Duration of disease (d, x ± s)	40.1 ± 15.68	42.7 ± 15.33	0.249
Comorbidities (n, %)	54 (75.0%)	50 (71.4%)	0.631
Hypertension	27 (37.5%)	20 (28.6%)	0.258
Diabetes	10 (13.9%)	11 (15.7%)	0.759
Hyperlipidemia	23 (31.9%)	21 (30.0%)	0.802
Symptoms (n, %)			
Shortness of breath	47 (65.3%)	43 (61.4%)	0.634
Cough	20 (27.8%)	25 (35.7%)	0.310
Fatigue	34 (47.2%)	32 (45.7%)	0.857
Insomnia	37 (51.4%)	38 (54.3%)	0.730
Sweating	30 (41.7%)	29 (41.4%)	0.977
Poor appetite	10 (13.9%)	8 (11.4%)	0.660
Diarrhea	10 (13.9%)	11 (15.7%)	0.759
6MWT (m, x ± s)	454.2 ± 65·68	465.2 ± 69.13	0.332
QOL-BREF score			
Subjective perception of quality of life	3.4 ± 0.87	3.5 ± 0.65	
Subjective feeling of health status	2.8 ± 0.94	3.0 ± 0.90	0.212
Physical health	57.6 ± 16.61	63.0 ± 12.99	0.033
Psychological	64.2 ± 16.60	67.0 ± 12.66	0.261
Social relationships	64.3 ± 16.56	65.3 ± 11.10	0.657
Environment	64.7 ± 15.79	68.7 ± 10.30	0.078
PHQ-9 score	5.2 ± 4.47	4.9 ± 4.49	0.682
GAD-7 score	3.3 ± 3.96	2.9 ± 3.39	0.514

BMI, body mass index; COPD, chronic obstructive pulmonary disease; CAHD, coronary atherosclerotic heart disease; 6MWT, 6-min walk test; QOL-BREF, quality of life-BREF (WHOQOL-BREF); PHQ, patient health questionnaire; GAD, generalized anxiety disorder; FZHY, fuzheng huayu.

### Primary outcomes

**FIGURE 2:**
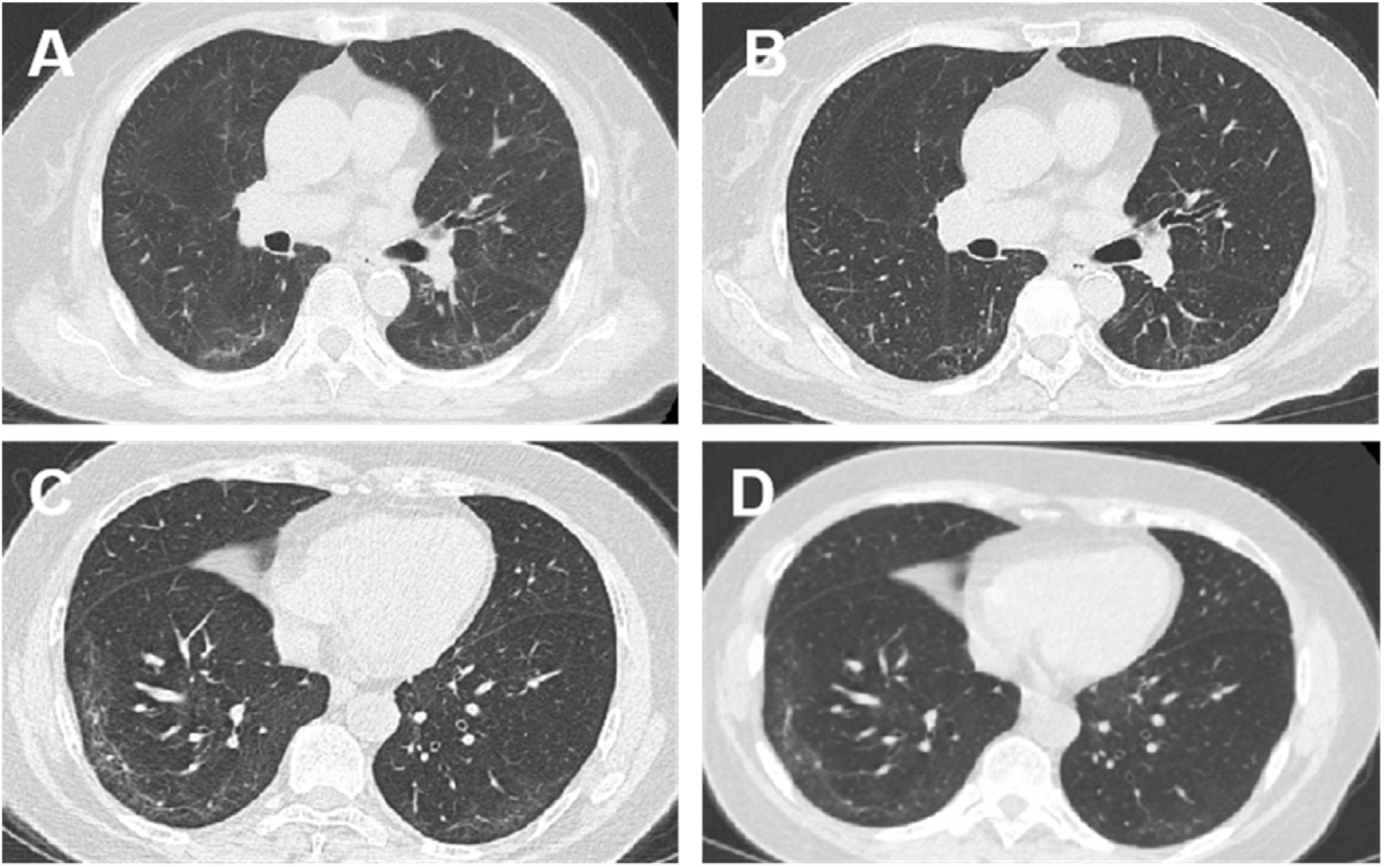
Chest computed tomography (CT) manifestations at enrolment and after intervention: CT findings of patients in the FZHY group and the placebo group. **(A)** Bilateral pulmonary infiltrates (more prominent in the right lower lobe) in a 70-year-old woman at initiation of FZHY treatment. **(B)** Marked absorption of bilateral pulmonary infiltrates 6 months after treatment with FZHY. **(C)** Bilateral pulmonary infiltrates in a 67-year-old woman at the initiation of placebo treatment. **(D)** No absorption of infiltrations in the bilateral lobes 6 months after the placebo treatment.

At baseline, HRCT scores for pulmonary fibrosis were comparable between the FZHY and placebo groups. At week 24, both groups exhibited a decrease in HRCT scores; however, the reduction was more significant in the FZHY group (−1.250 ± 1.780) than in the placebo group (−0.450 ± 1.354, p = 0.005, Supplementary Tables S5, S6). After 24 weeks of treatment, the regression rate of pulmonary fibrosis was higher in the FZHY group than in the placebo group (71.2% [47/66] vs 49.2% [31/63]; p = 0.011) (Table 2). Figure 2 shows the chest HRCT findings of the patients in the FZHY and placebo groups.

**TABLE 2:** Regression rates of pulmonary fibrosis.

	FZHY group	Placebo group	*p*-value
Regression rate of pulmonary fibrosis (n, %, FAS)	48/72 (66.7)	33/70 (47.1)	0.019
Regression rate of pulmonary fibrosis (n, %, PPS)	47/66 (71.2)	31/63 (49.2)	0.011

PPS, per protocol set; FAS, full analysis set; FZHY, fuzheng huayu.

**FIGURE 3:**
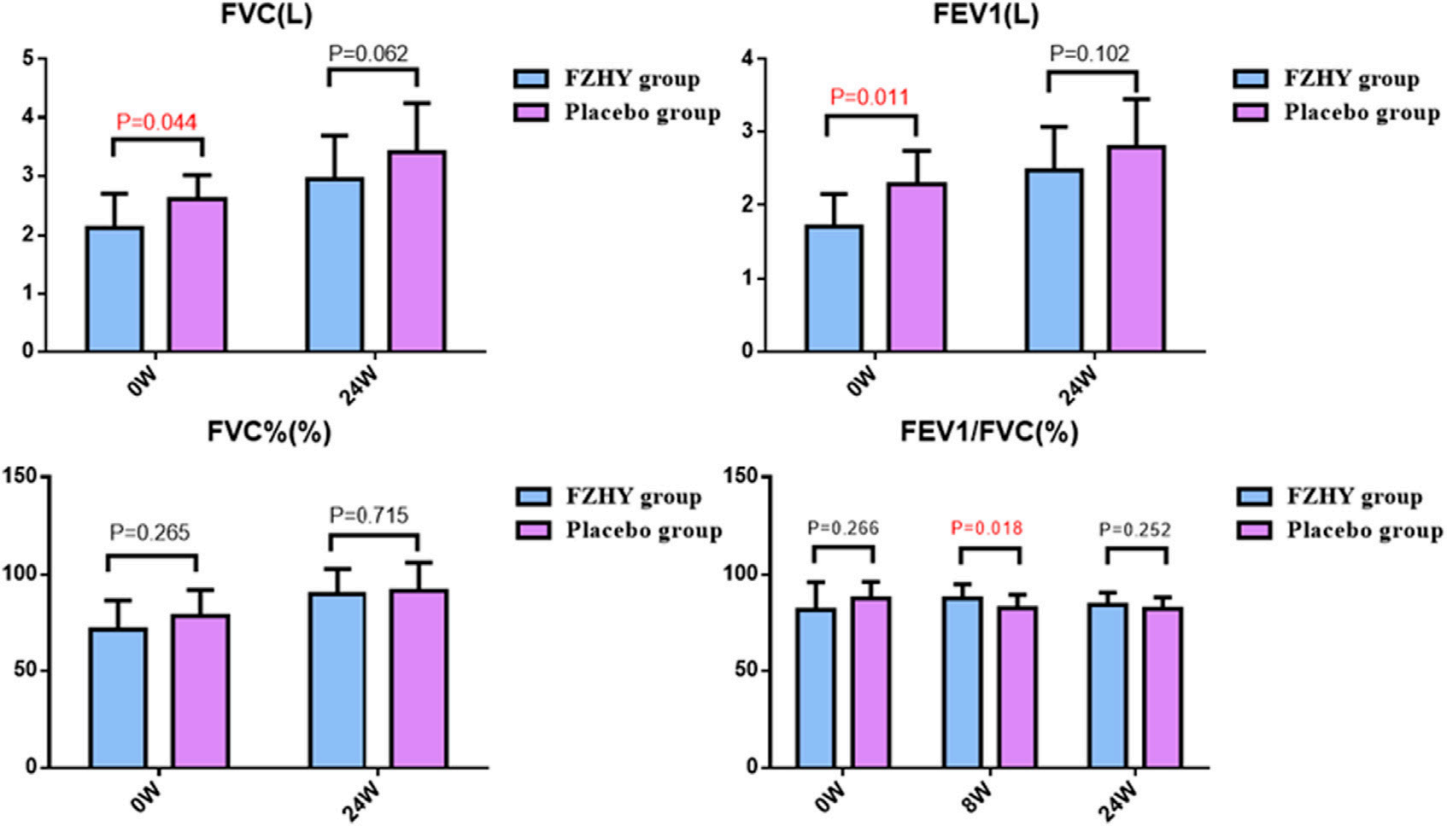
Pulmonary function manifestations at enrolment and after treatment. FVC: Forced vital capacity; FEV1: Forced expiratory volume in one second.

A spirometer was used in a few patients at only one center, while diffusing capacity assessments were not performed to minimize the risk of cross-infection during emergencies. At baseline, both FVC and FEV1 were lower in the FZHY group than in the placebo group (FZHY group, n = 9; placebo group, n = 11). After 24 weeks of treatment, no significant difference in FVC or FEV1 was observed between the two groups (FZHY group, n = 21; placebo group, n = 22). However, the FEV1/FVC ratio was comparable between the FZHY and placebo groups at baseline. At week 8, a significant difference in FEV1/FVC was observed between the FZHY group ([87.7 ± 7.2] %, n = 22) and the placebo group ([82.7 ± 6.9] %, n = 25) (p = 0.018). These results suggest that FZHY may promote the recovery of pulmonary ventilation during the early stages of post-COVID-19 pulmonary fibrosis (Figure 3).

### Secondary outcomes

#### Six-minute walk distance

**FIGURE 4:**
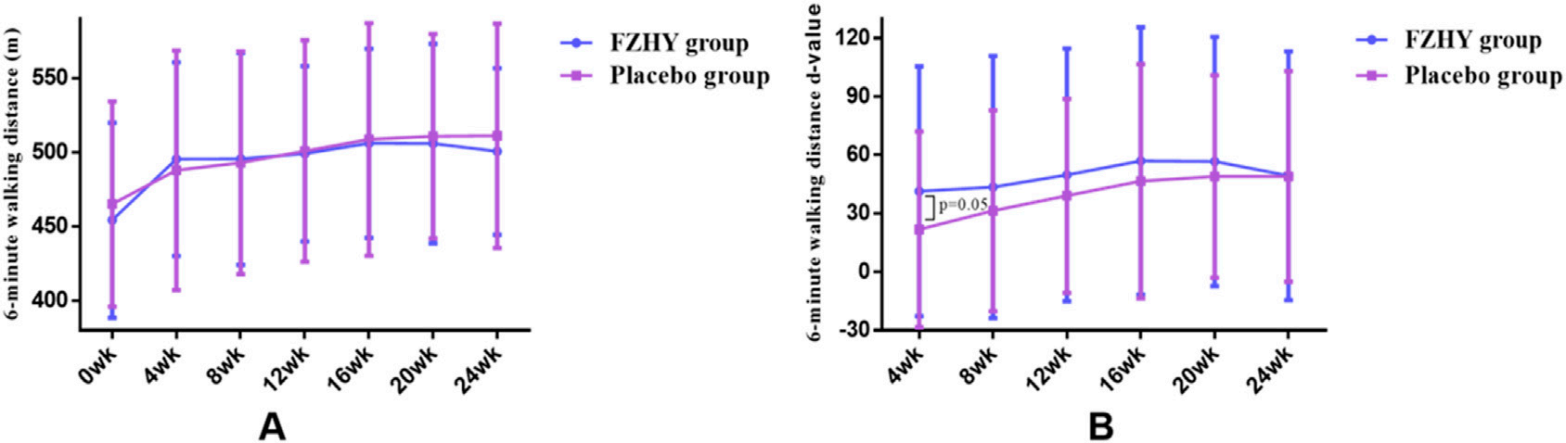
**(A)** Dynamic changes in 6-min walking distance in both groups. **(B)** Six-minute walk distance d-value in both groups.

After 24 weeks of treatment, both the FZHY and placebo groups demonstrated a trend toward improved performance in the 6-min walk test; however, this difference was not statistically significant when comparing the two groups at each visit (Figure 4A). However, the increase in 6-min walk distance at week four was statistically significant, with the FZHY group showing a substantial increase than that of the placebo group ([41.4 ± 64.1] m vs [21.8 ± 50.3] m, p = 0.05; Figure 4B), suggesting that FZHY may promote recovery of lung function in the early stage of post-COVID-19 pulmonary fibrosis.

#### Regression rate of lung inflammation

After 24 weeks of treatment, the rate of improvement in lung inflammation was significantly higher in the FZHY group than in the placebo group (83.8% vs 68.8%, p = 0.041, Table 3).

**TABLE 3:** Regression rate of lung inflammation (Full analysis set).

	FZHY group (n = 68)	Placebo group (n = 64)	*p*-value
Regression rate of lung inflammation (n, %)	57 (83.8)	44 (68.8)	0.041

FZHY, fuzheng huayu.

#### Disappearance rate of clinical symptoms

No significant difference was observed in the rate of disappearance of clinical symptoms between the two groups after 24 weeks of treatment (Table 4). However, some data was missing, which might have affected the accuracy of the results.

**TABLE 4:** Disappearance rate of clinical symptoms (Full analysis set).

	FZHY group	Placebo group	*p*-value
Shortness of breath (n, %)	34 (81.0) (n = 42)	33 (80.5) (n = 41)	0.957
Cough (n, %)	15 (83.3) (n = 18)	21 (91.3) (n = 23)	0.638
Fatigue (n, %)	19 (63.3) (n = 33)	21 (72.4) (n = 29)	0.456
Insomnia (n, %)	21 (63.6) (n = 33)	21 (55.3) (n = 38)	0.474
Sweating (n, %)	23 (79.3) (n = 29)	23 (82.1) (n = 28)	0.786
Poor appetite (n, %)	9 (100.0) (n = 9)	7 (100·0) (n = 7)	NA
Diarrhea (n, %)	8 (88.9) (n = 9)	11 (100.0) (n = 11)	0.450

Clinical symptom disappearance rate = (number of persons with the symptom present at baseline - number of persons with the symptom present at 24 weeks)/number of persons with the symptom present at baseline. FZHY, fuzheng huayu.

#### QOL-BREF, PHQ-9, and GAD-7 scores

After 24 weeks of treatment, no significant differences in QOL were observed between the two groups (Table 5).

**TABLE 5:** Comparison of the QOL between the two groups at week 24 (Full analysis set).

		FZHY group (n = 63)	Placebo group (n = 62)	*p*-value
QOL-BREF score	Subjective perception of quality of life	3.7 ± 0.65	3.7 ± 0.56	0.737
	Subjective feeling of health status	3.3 ± 0.86	3.3 ± 0.78	0.642
	Physical health	69.8 ± 13.59	68.4 ± 10.51	0.542
	Psychological	70.5 ± 15.98	71.1 ± 12.30	0.813
	Social relationships	63.2 ± 15.26	65.0 ± 10.40	0.452
	Environment	67.5 ± 12.60	69.1 ± 10.29	0.442
PHQ-9 score		2.7 ± 4.05	2.6 ± 2.67	0.807
GAD-7 score		2.3 ± 3.79	1.6 ± 2.37	0.214

FZHY, fuzheng huayu; QOL-BREF, quality of life-BREF; PHQ, patient health questionnaire; GAD, generalized anxiety disorder.

#### Harms

AEs such as abnormal liver function tests, abnormal electrocardiogram findings, and increased urinary leukocytes were observed in both the FZHY and placebo groups. In addition, renal dysfunction was reported in two patients, and dyslipidemia and hyperglycemia were reported in one patient each in the FZHY group. In the placebo group, increased C-reactive protein levels, leukocytosis, and back pain were reported in one patient each. However, it was later confirmed that none of these AEs were related to the trial. No significant differences in AEs were observed between the two groups (p > 0.05, Table 6). Notably, no serious AEs were reported in either group.

**TABLE 6:** Comparison of the adverse events in the full analysis set (safety analysis set).

	FZHY group (n = 72)	Placebo group (n = 70)	*p*-value
Abnormal liver function (n, %)	1 (1.4)	3 (4.3)	0.363
Renal dysfunction (n, %)	2 (2.8)	0 (0)	0.497
Abnormal ECG (n, %)	6 (8.3)	3 (4.3)	0.494
Urinary leukocytosis (n, %)	4 (5.6)	5 (7.1)	0.743
Dyslipidemia (n, %)	1 (1.4)	0 (0)	1.000
Hyperglycemia (n, %)	1 (1.4)	0 (0)	1.000
Increased C-reactive protein (n, %)	0 (0)	1 (1.4)	0.493
Leukocytosis (n, %)	0 (0)	1 (1.4)	0.493
Back pain (n, %)	0 (0)	1 (1.4)	0.493

FZHY, fuzheng huayu; ECG, electrocardiogram.

## Discussion

Our findings indicated that after 24 weeks of treatment, the regression rate of pulmonary fibrosis was 71.2% and 49.2% in the FZHY and placebo groups, respectively (p = 0.011). Limited spirometry data revealed FEV1/FVC was higher in the FZHY group than in the control group (87.7 ± 7.2 vs 82.7 ± 6.9) after 8 weeks of treatment. The improvement in pulmonary inflammation was 83.8% in the FZHY group and 68.8% in the placebo group (p < 0.05). The increase in 6-min walking distance was higher in the FZHY group than in the control group (41.4 ± 64.1 vs 21.8 ± 50.3) at week 4, with no difference thereafter. No drug-related AEs were observed in the FZHY group.

We defined convalescence in COVID-19 as the stage when the patient meets the discharge criteria for clinical cure, yet many clinical symptoms persisted. During this phase, the patient’s functional abilities may not be fully restored, and they may experience immune system disorders, along with other challenges, including respiratory issues, physical and psychological problems, difficulties in daily living, and limitations in social participation (Wen et al., 2020). Our findings indicated that partially recovered COVID-19 patients still exhibited associated abnormalities on chest CT imaging, including ground-glass opacities, grid shadowing, and fibrous banding during the recovery period. Notably, FZHY promoted the absorption of fibrotic lesions after inflammation during the recovery period in COVID-19 patients.

Radiographic findings indicated improvement in pulmonary fibrosis after 24 weeks, as assessed by manual semi-quantitative scoring of chest CT in convalescent COVID-19 patients, with an improvement rate of 71.2% in the FZHY group and 49.2% in the placebo group. Furthermore, lung inflammation was significantly improved after 24 weeks, with an improvement rate of 83.8% and 68.8% in the FZHY and placebo groups, respectively. This study was conducted at the time of epidemic ravaging, when pulmonary function testing was not performed on patients in most centers to avoid cross-infection. Nevertheless, the limited spirometry results showed a possible effect of FZHY on improving lung function in the early stages of recovery.

In addition to causing pulmonary fibrosis, COVID-19 can lead to various functional impairments, such as fatigue, insomnia, and anxiety, in patients during the recovery phase (Huang et al., 2021). Xiong et al. (2021) observed that COVID-19 patients experienced increased resting heart rate, palpitations, elevated blood pressure, and other psychiatric symptoms such as sleep disturbances, anxiety, irritability, low self-esteem, and depression 3 months after hospital discharge. Carda et al. (2020) found that COVID-19 patients had sequelae such as cognitive impairment, peripheral nervous system damage, and psychiatric problems. Li et al. (2020) found that SARS-CoV-2 was still present in the semen of some COVID-19 patients who had achieved clinical recovery. Additionally, this study reported that COVID-19 patients often continued to experience clinical manifestations such as cough, chest tightness, and fatigue after discharge, along with emotional distress, including anxiety and depression. Regrettably, in this study, compared with the placebo group, the FZHY group failed to effectively improve these symptoms. FZHY is mainly used to treat fibrotic diseases, particularly liver fibrosis, and is not specific for symptom manifestations in the respiratory system, which may explain its limited effect on symptoms such as cough and chest tightness. Conversely, the limited number of observations for some variables may have constrained our ability to detect significant differences, potentially leading to selection biases. Despite these limitations, our study highlighted that QOL-BREF, PHQ-9, and GAD-7 scores significantly improved in both placebo and FZHY groups after 24 weeks of treatment.

Theoretical and research findings demonstrate the potential of FZHY in improving post-inflammatory pulmonary fibrosis. Our previous results have shown that this formula regulates immune and inflammatory responses (Chen et al., 2019; Zhang et al., 2020). Patients with COVID-19 often present with numerous underlying conditions and may themselves have an immune imbalance (Huang et al., 2020; Biswas, 2016). Notably, most patients included in this study were middle-aged or older, and more than 70% of patients had concomitant underlying diseases. Multiple active ingredients in FZHY exhibit anti-inflammatory and anti-pulmonary fibrotic effects. The most important active component of Danshen is Tanshinone ⅡA. Feng et al. (2020) found that Tanshinone IIA can partially ameliorate fibrosis *in silico* by inhibiting the activation of the TGF-β/Smad signaling pathway. He et al. (2015) found that Tanshinone IIA reduced inflammatory cell infiltration, proinflammatory cytokine release, and collagen deposition in BLM-induced rats. Furthermore, An et al. (2019) found that Tanshinone IIA activated nuclear factor erythroid 2-related factor 2 by regulating the redox balance and glutaminolysis, thereby inhibiting pulmonary fibrosis. In addition, Chen et al. (2012) demonstrated the potential of Cordyceps to prevent and treat fibrosis by decreasing inflammatory cell infiltration, fibroblast and collagen deposition, and restoring the MMP-9/TIMP-1 imbalance in rats with pulmonary fibrosis. Xu et al. (2011) found that Cordyceps sinensis decreased CTGF, hydroxyproline, and TGF-β1 expression in pulmonary fibrosis rats, with enhanced antifibrotic effects when combined with glucocorticoids. Zhai et al. (2014) found that ephedrine combined with Schisandra mitigated the development of alveolar inflammation and fibrosis in rats with BLM-induced pulmonary fibrosis. Schisandra exerted anti-fibrotic effects by inhibiting M2 macrophage polarization in BLM-induced pulmonary fibrosis in rats (Guo et al., 2020).

This study had several limitations. First, the majority of patients in this study were enrolled between April and August 2020. During this period, none of the participants had received the COVID-19 vaccine, and the predominant strain was the wild-type SARS-CoV-2, which differs from subsequent variants. Secondly, because COVID-19 is an emerging disease, the literature was limited for sample size determination, and the sample size was not adjusted since the wile type COVID−19 epidemic was quickly diminished during the implementation of this study. Third, the chest CT scoring in this study used an artificial semi-quantitative method, which may introduce subjective bias in the results. Additionally, spirometry was performed in only a limited number of patients to avoid cross-infection, and tests for diffusion indicators were not available in the study for assessing the anti-lung fibrotic efficacy.

Given that pulmonary fibrosis is a chronic and progressive condition with multiple etiologies, it is imperative to conduct multicenter trials with larger sample sizes and extended treatment durations to reconfirm the efficacy of FZHY on pulmonary fibrosis caused by various etiologies. Nevertheless, our current data warrants further investigation for FZHY on the efficacy with different etiologies and action mechanisms in depth against pulmonary fibrosis.

## Conclusion

FZHY tablets attenuate pulmonary fibrosis and inflammation in convalescent patients with COVID-19, with promising safety profiles.

## Data Availability

The original contributions presented in the study are included in the article/Supplementary Material, further inquiries can be directed to the corresponding authors.

## Abbreviations

BLM, bleomycin; COPD, chronic obstructive pulmonary disease; COVID-19, Coronavirus Disease 2019; FEV1, forced expiratory volume in the first second; FVC, forced vital capacity; FAS, full analysis set; FZHY, Fuzheng Huayu formula; HRCT, high-resolution computed tomography; IWRS, interactive web randomization system; PPS, per protocol set; RT-PCR, real-time fluorescence quantitative polymerase chain reaction; SARS, severe acute respiratory syndrome.
