# Supplementary material for: Fuzheng Huayu tablets for treating pulmonary fibrosis in post-COVID-19 patients: a multicenter, randomized, double-blind, placebo-controlled trial
Source: Front Pharmacol. 2025 Mar 11;16:1508276. doi: 10.3389/fphar.2025.1508276 (PMC11933019; doi:10.3389/fphar.2025.1508276)
Supplement: Supplementary file 1 [file DataSheet1.docx]

**Supplementaries**

**Formulation of Fuzheng Huayu Tablet**

All the raw materials for FZHY comes from the medicinal material planting and harvesting bases according to Good Agricultural and Collection Practices (GACP) requirement in definite places, and identified for their origin, morphological, microscopic, physical, chemical characteristics, as well as the DNA barcode identification in order to guarantee the authenticity and quality of medicinal materials.

References:

Hassanein, T., Tai, D., Liu, C., Box, T. D., Tong, M. J., Rossaro, L., Pozza, R., Glenn, J. S., Cheung, R., Hemaidan, A., He, Y., Behling, C., Hu, X., Makhlouf, H., Fan, H., Ren, Y., Khim Chng, E. L., Liu, P., Vierling, J. M., 2022. Efficacy and Safety of a Botanical Formula Fuzheng Huayu for Hepatic Fibrosis in Patients with CHC: Results of a Phase 2 Clinical Trial. Evidence-based complementary and alternative medicine: eCAM, 2022, 4494099.

**Table S1 Fuzheng Huayu (FZHY) formulation (g/ daily dose).**

| **Chinese name** | **Plant sources** | **Medicinal parts** | **Preparation amount (g)** |
| --- | --- | --- | --- |
| Danshen | Salvia Miltiorrhizae Bge (Labiatae) | radix | 8 |
| Chongcao | artificial fermentation cordyceps | mycelia | 4 |
| Taoren | Prunus persica (L.) Batsch (Rosaceae) | fruit | 2 |
| Jiaogulan | Gynostemma pentaphyllum (Thunb) | whole herb | 6 |
| Songhuafen | Pinus massoniana Lamb (Pinaceae) | pollen | 2 |
| Wuweizi | Schisandrae Chinensis (Turcz.)Baill | fruit | 2 |

**Quality Control** **of Fuzheng Huayu Tablet**

**Standard Manufacturing Process**

For the preparation of FZHY extraction, 666g of Danshen, 500g of Jiaogulan, 334g of Chongcao, 166g of each, Taoren, Songhuafen and Wuweizi are weighed up. Danshen, Taoren and Jiaogulan are first mixed with appropriate amount of water to decoct twice, 2 hours for the first time and 1.5 hour for the second time. The decoctions are then combined and left to stand for 24h to allow the supernatant to concentrate until a relative density of about 1.20 (50–55℃). This is then cooled, and alcohol (95%) is added while slowly shaking up until the alcohol content reaches 70%. It is then cooled again, filtrated and concentrated until the relative density reaches 1.3–1.4 at 50–55℃. Drying takes place by decompression to obtain the dry extract. Then, Chongcao and Wuweizi are weighed up, reflux extraction with alcohol twice, 2 hours for the first time and 1.5 hour at the second time. The extract solution is combined, filtrated and concentrated until the relative density reaches 1.3–1.4 at 50–55℃. The alcohol is recovered and dried by decompression to get the dry extract. Similarly, Songhuafen is soaked in 50% alcohol at 40℃ twice, 4 hours for the first time and 2 hours for the second time. The extract solution is combined, filtrated and concentrated until the relative density reaches 1.3–1.4 at 50–55℃ The alcohol is recovered , dried and decompressed to obtain the dry extract. Those three dry extracts are mixed and dried as the FZHY extract powder to reserve.

Then pharmaceutical excipients are added to the FZHY extract powder and the tablets are prepared directly by compression.

It can be compressed to 1000 tablets (0.4g each) or 500 tablets (0.8g each).

**
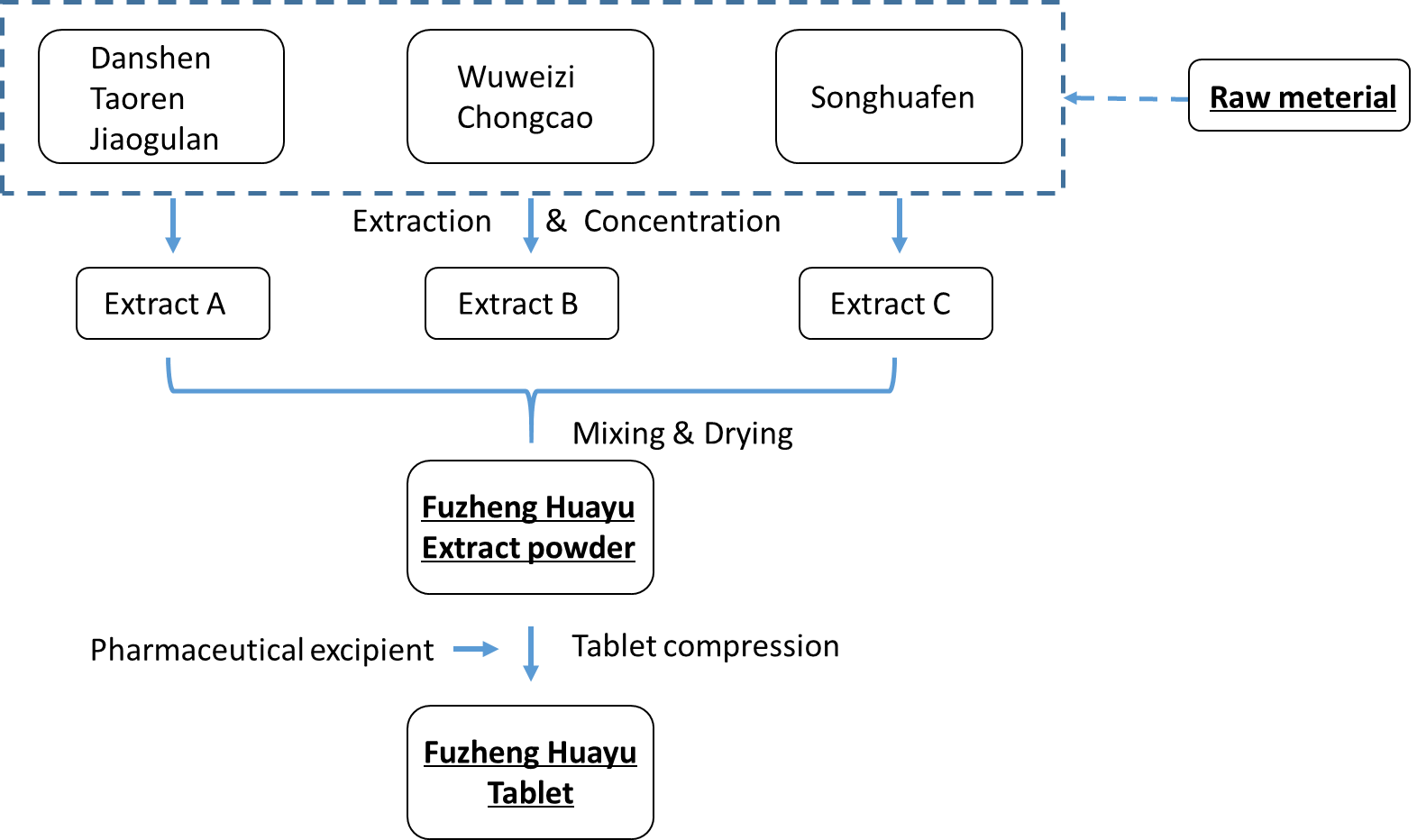
**

**Fig. S1.** General manufacturing protocol for FZHY tablet.

**Table S2 Chemical Components Content of FZHY.**

| **Compounds (marker)** | **Quality criterion** |
| --- | --- |
| Salvianolic acid B  (from Danshen) | Should be no less than 15.6mg in 24g of FZHY raw materials (daily dose） |
| Sodium Danshensu  (from Danshen) | Should be no less than 13.2mg in 24g of FZHY raw materials (daily dose） |
| Adenosine  (from Chongcao) | Should be no less than 4.8mg in 24g of FZHY raw materials (daily dose) |
| Schisandrin B  (from Wuweizi) | Should be no less than 2.28mg in 24g of FZHY raw materials (daily dose) |

**Component markers standard for FZHY**

**Multi-components assay (Fingerprinting)**

To control the quality of the FZHY extracts, the fingerprint spectrum was established using high performance liquid chromatography (HPLC) method. Assay validation was performed according to the United States Food and Drug Administration (US FDA) bio-analytical method validation guideline (FDA, Center for Drug Evaluation & Research, 2001). The chromatographic proﬁle of the extracts is shown in Figure 2. The contents of adenosine and danshensu were 2.5mg/g and 8.04mg/g in the extracts respectively, according to quality inspection report from the Shanghai Sundise Chinese Medicine Technology Development Co., Ltd (Shanghai, China).


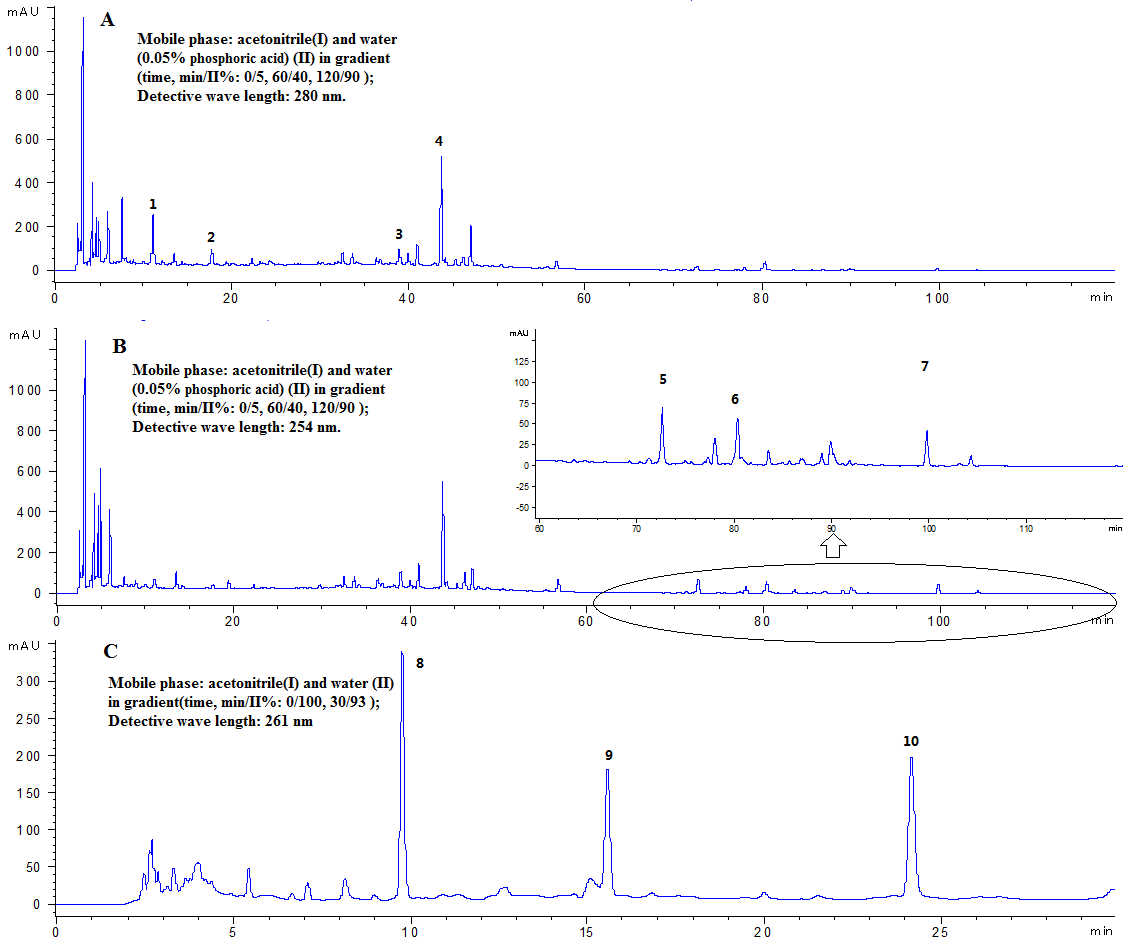


**Fig. S2.** The chromatographic proﬁle of FZHY extracts [Stationary phase: CNW Athena C18-WP (4.6mm×150mm, 3µm), ﬂow rate: 1 mL/min). Peak No.: 1. danshensu; 2. protocatechuic aldehyde; 3. rosmarinic acid; 4. salvianolic acid B; 5. schizandrol A; 6. schizandrol B; 7. schizandrin A; 8. uridine 9. guanosine; 10. adenosine.**Distribution of participants in each center**

**Table S3 Distribution of Participants in Each Center**

|  | **FZHY group** | **Placebo group** | Total |
| --- | --- | --- | --- |
| Wuhan Integrated TCM & Western Medicine Hospital | | | |
| Randomized | 39 | 36 | 75 |
| Completed | 34 | 32 | 66 |
| FAS | 39 | 36 | 75 |
| PPS | 34 | 32 | 66 |
| SS | 39 | 36 | 75 |
| Hubei Provincial Hospital of Traditional Chinese Medicine | | | |
| Randomized | 15 | 19 | 34 |
| Completed | 15 | 18 | 33 |
| FAS | 15 | 19 | 34 |
| PPS | 15 | 18 | 33 |
| SS | 15 | 19 | 34 |
| Huangshi Hospital of Traditional Chinese Medicine | | | |
| Randomized | 9 | 7 | 16 |
| Completed | 9 | 6 | 15 |
| FAS | 9 | 7 | 16 |
| PPS | 9 | 6 | 15 |
| SS | 9 | 7 | 16 |
| Wuhan Third Hospital | | | |
| Randomized | 7 | 6 | 13 |
| Completed | 7 | 6 | 13 |
| FAS | 7 | 6 | 13 |
| PPS | 7 | 6 | 13 |
| SS | 7 | 6 | 13 |
| Jingmen First People's Hospital | | | |
| Randomized | 2 | 2 | 4 |
| Completed | 1 | 1 | 2 |
| FAS | 2 | 2 | 4 |
| PPS | 1 | 1 | 2 |
| SS | 2 | 2 | 4 |
| Total | | | |
| Randomized | 72 | 70 | 142 |
| Completed | 66 | 63 | 129 |
| FAS | 72 | 70 | 142 |
| PPS | 66 | 63 | 129 |
| SS | 72 | 70 | 142 |

**Concomitant drug use among participants**

**Table S4 Analysis of concomitant medications (ATC classification) during treatment**

| **ATC First Level classification  ATC second level classification  Preferred term** | **FZHY group** | **Placebo group** |
| --- | --- | --- |
| N | 72 | 70 |
| Summary n(%) | 4(5.6) | 9(12.9) |
| Cardiovascular system n(%) | 3(4.2) | 6(8.6) |
| Calcium channel blockers n(%) | 3(4.2) | 4(5.7) |
| Dihydropyridine derivative n(%) | 1(1.4) | 1(1.4) |
| Felodipine n(%) | 1(1.4) | 1(1.4) |
| Nifedipine n(%) | 1(1.4) | 1(1.4) |
| Amlodipine n(%) | 0(0) | 1(1.4) |
| Drugs acting on the renin-angiotensin system n(%) | 1(1.4) | 1(1.4) |
| Captopril n(%) | 1(1.4) | 0(0) |
| Valsartan and amlodipine n(%) | 0(0) | 1(1.4) |
| Beta-blocker n(%) | 1(1.4) | 0(0) |
| Bisoprolol n(%) | 1(1.4) | 0(0) |
| Diuretic n(%) | 0(0) | 1(1.4) |
| Hydrochlorothiazide n(%) | 0(0) | 1(1.4) |
| Vascular protectant n(%) | 0(0) | 1(1.4) |
| Calcium Dobesilate n(%) | 0(0) | 1(1.4) |
| Lipid regulator n(%) | 0(0) | 1(1.4) |
| Atorvastatin and aspirin n(%) | 0(0) | 1(1.4) |
| Digestive tract and metabolism n(%) | 2(2.8) | 2(2.9) |
| Diabetes drugs n(%) | 2(2.8) | 1(1.4) |
| Acarbose n(%) | 1(1.4) | 1(1.4) |
| Metformin n(%) | 1(1.4) | 0(0) |
| Insulin glargine n(%) | 0(0) | 1(1.4) |
| Gliclazide n(%) | 1(1.4) | 0(0) |
| Drugs for liver and gallbladder diseases n(%) | 0(0) | 1(1.4) |
| Hepatotherapy n(%) | 0(0) | 1(1.4) |
| Non-sex hormone and insulin hormone system drugs n(%) | 0(0) | 1(1.4) |
| Thyroid therapeutics n(%) | 0(0) | 1(1.4) |
| Levothyroxine sodium n(%) | 0(0) | 1(1.4) |
| Respiratory system n(%) | 0(0) | 1(1.4) |
| Drugs for obstructive tracheal diseases n(%) | 0(0) | 1(1.4) |
| Salmeterol combined with fluticasone n(%) | 0(0) | 1(1.4) |

**Table S5 Analysis of HRCT scores of pulmonary fibrosis (FAS)**

|  | **FZHY group** | **Placebo group** | ***P*value** |
| --- | --- | --- | --- |
| 0wk | | | |
| N | 72 | 70 | 0.989 |
| Mean±SD | 5.775±5.701 | 5.762±6.203 |  |
| 95%CI | 4.436～7.115 | 4.283～7.241 |  |
| 24wk | | | |
| N | 72 | 70 | 0.417 |
| Mean±SD | 4.606±4.994 | 5.357±5.963 |  |
| 95%CI | 3.433～5.780 | 3.935～6.779 |  |
| 24wk-0wk | | | |
| N | 72 | 70 | 0.003 |
| Mean±SD | -1.169±1.734 | -0.405±1.291 |  |
| 95%CI | -1.576～-0.762 | -0.712～-0.097 |  |

**Table S6 Analysis of HRCT scores of pulmonary fibrosis (PPS)**

|  | **FZHY group** | **Placebo group** | ***P*value** |
| --- | --- | --- | --- |
| 0wk | | | |
| N | 66 | 63 | 0.990 |
| Mean±SD | 6.111±5.824 | 6.098±6.399 |  |
| 95%CI | 4.679～7.543 | 4.486～7.709 |  |
| 24wk | | | |
| N | 66 | 63 | 0.431 |
| Mean±SD | 4.861±5.117 | 5.648±6.163 |  |
| 95%CI | 3.603～6.119 | 4.096～7.200 |  |
| 24wk-0wk | | | |
| N | 66 | 63 | 0.005 |
| Mean±SD | -1.250±1.780 | -0.450±1.354 |  |
| 95%CI | -1.688～-0.812 | -0.791～-0.109 |  |
